# An Obligate Role of Oxytocin Neurons in Diet Induced Energy Expenditure

**DOI:** 10.1371/journal.pone.0045167

**Published:** 2012-09-18

**Authors:** Zhaofei Wu, Yuanzhong Xu, Yaming Zhu, Amy K. Sutton, Rongjie Zhao, Bradford B. Lowell, David P. Olson, Qingchun Tong

**Affiliations:** 1 Brown Foundation Institute of Molecular Medicine, University of Texas Medical School at Houston, Houston, Texas, United States of America; 2 Graduate Programs in Biochemistry and Neuroscience of Graduate School of Biomedical Sciences, University of Texas Medical School at Houston, Houston, Texas, United States of America; 3 Division of Endocrinology, Beth Israel Deaconess Medical Center and Harvard Medical School, Boston, Massachusetts, United States of America; 4 Departments of Pediatrics and Molecular and Integrative Physiology, University of Michigan, Ann Arbor, Michigan, United States of America; University of Texas Health Science Center at San Antonio, United States of America

## Abstract

Oxytocin neurons represent one of the major subsets of neurons in the paraventricular hypothalamus (PVH), a critical brain region for energy homeostasis. Despite substantial evidence supporting a role of oxytocin in body weight regulation, it remains controversial whether oxytocin neurons directly regulate body weight homeostasis, feeding or energy expenditure. Pharmacologic doses of oxytocin suppress feeding through a proposed melanocortin responsive projection from the PVH to the hindbrain. In contrast, deficiency in oxytocin or its receptor leads to reduced energy expenditure without feeding abnormalities. To test the physiological function of oxytocin neurons, we specifically ablated oxytocin neurons in adult mice. Our results show that oxytocin neuron ablation in adult animals has no effect on body weight, food intake or energy expenditure on a regular diet. Interestingly, male mice lacking oxytocin neurons are more sensitive to high fat diet-induced obesity due solely to reduced energy expenditure. In addition, despite a normal food intake, these mice exhibit a blunted food intake response to leptin administration. Thus, our study suggests that oxytocin neurons are required to resist the obesity associated with a high fat diet; but their role in feeding is permissive and can be compensated for by redundant pathways.

## Introduction

The paraventricular hypothalamus (PVH) is a critical brain region for both feeding and energy expenditure regulation [1]–[2]. Within the PVH, there are distinct subsets of peptidergic neurons including oxytocin, vasopressin (AVP), thyrotropin releasing hormone (TRH) and corticotropin releasing hormone (CRH) neurons, which send projections throughout the brain as well as to the median eminence (TRH and CRH) or posterior pituitary (oxytocin and vasopressin, also including projections from the supraoptic nucleus) [3]. These projections form the structural basis through which the PVH in the regulates a diverse set of physiologic functions including energy homeostasis.

Substantial data supports a role for oxytocin in regulating body weight. Oxytocin neurons show relatively high co-localization with the expression of *FTO* gene, a gene in which mutations have been shown to be significantly associated with human obesity [4]. Reduced oxytocin neuron number and cell volume, and reduced baseline oxytocin profiles have been associated with the Prader-Willi syndrome, a human obesity syndrome notable for severe hyperphagia [5]–[6]. Oxytocin neurons appear to at least partially mediate the anorexigenic action of leucine [7]. Administration of oxytocin decreases food intake while administration of oxytocin receptor antagonists results in hyperphagia [8]. Current evidence supports a model in which PVH oxytocin neurons project to the nucleus of solitary tract (NTS) and release oxytocin to modulate the activity of local hindbrain neurons and “fine tune” the response of NTS neurons to satiety signals arising in the gut and/or periphery [9]–[12]. In addition, diminished oxytocin has been shown to be associated with hyperphagic obesity secondary to haploinsufficiency of *Single-minded 1*, a transcription factor required for PVH development [13]. Importantly, oxytocin reduces high-fat induced obesity by restricting energy intake [14]–[15]. Consistent with this result, a recent study suggested that synaptotagmin-4 regulates oxytocin release to modulate feeding and that defects in this regulation may mediate diet-induced obesity [8], [16]. Taken together, these data demonstrate an important role for oxytocin in the regulation of food intake.

Despite the compelling evidence for a role of oxytocin in feeding regulation, there are inconsistencies regarding the role of oxytocin in other animal studies. Mice with deficiency of oxytocin or its receptor show either normal body weight or mild obesity [3], [17]–[20]. Even in the case of obesity, mice show reduced energy expenditure but normal feeding [17], [20]–[21]. Whether these discrepancies could be attributable to developmental compensation in response to germline gene deletion is not clear. Previous studies targeting agouti-related peptide (AgRP) neurons revealed that disruption of these neurons in the neonatal period induced profound developmental compensation that almost completely masked the physiologic function of these neurons [22]. To test the necessity of oxytocin neurons in feeding regulation and avoid any developmental compensation occurring from germline deletion of oxytocin or its receptor, we generated mice with a specific lesion of oxytocin neurons in adult mice using a temporally controlled, genetic lesioning approach. After achieving ∼ 95% ablation of oxytocin neurons, our results demonstrate that oxytocin neurons are dispensable for feeding regulation in males and females, but are required for diet induced energy expenditure and for pharmacologic leptin action on feeding in males.

## Materials and Methods

### Experimental Animals

*Oxytocin-Ires Cre* mice were generated using recombineering techniques as previously described [23]–[24]. Briefly, a selection cassette containing and internal ribosomal entry sequence linked to Cre recombinase and an Frt-flanked kanamycin resistance gene was targeted just downstream of the stop codon of the Oxytocin gene in a bacterial artificial chromosome (RP24-388N9; Children’s Hospital Oakland Research Institute). A targeting plasmid containing the Cre-containing selection cassette and 4 kb genomic sequence upstream and downstream of the Oxytocin stop codon was isolated and used for embryonic stem cell targeting. Correctly targeted clones were identified by long range PCR and southern blot analysis and injected into blastocysts. Chimeric animals generated from blastocyst implantation were then bred for germline transmission of the altered Oxytocin-allele. Flp-deleter mice were then used to remove the neomycin selection cassette.

**Figure 1:**
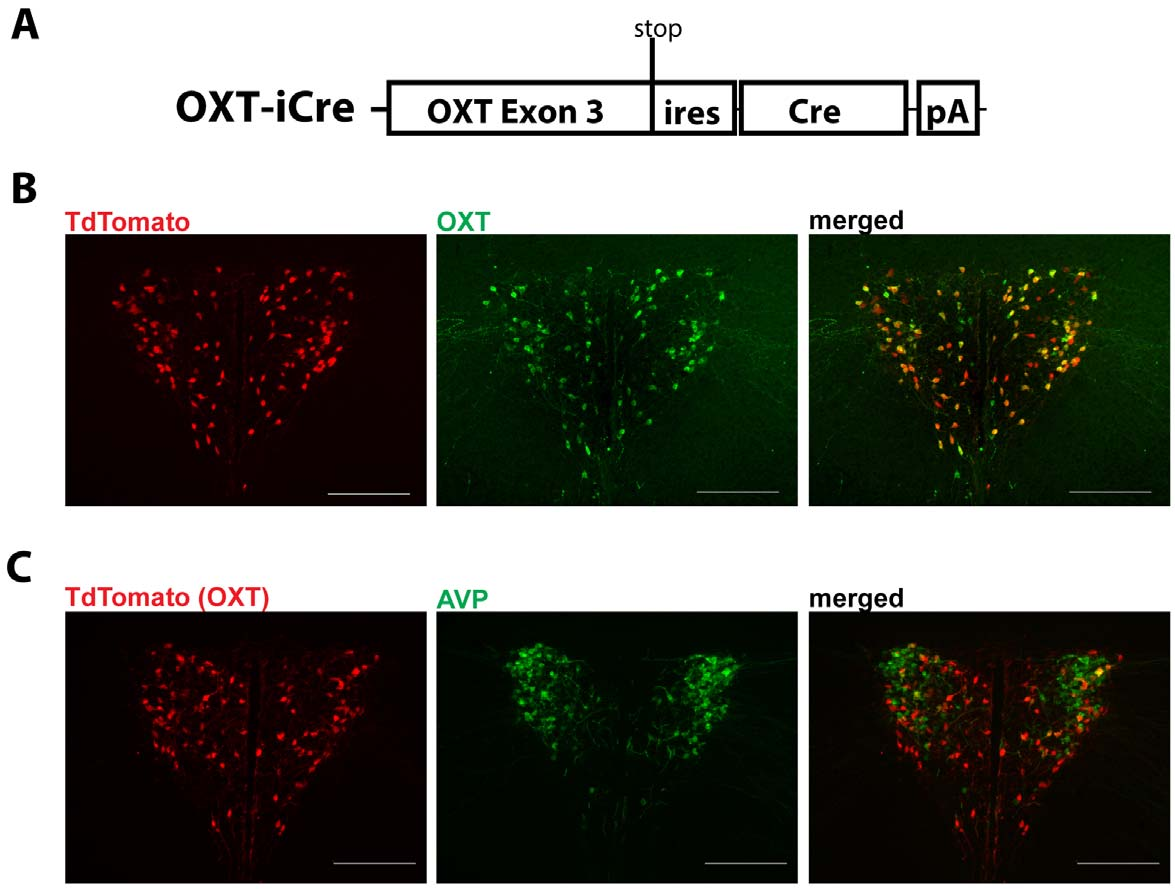
*Oxytoin-Ires-Cre* knock-in mice express Cre recombinase in oxytocin neurons. A) Cre recombinase was targeted just after the stop codon of the *Oxytocin* gene using an internal ribosomal entry site (ires). B) Immunohistochemistry for Oxytocin on brain slices of *Oxytocin-Ires-Cre*:Td-tomato reporter (Ai9) mice indicates that nearly all oxytocin-containing neurons (green) express Cre recombinase. C) Oxytocin-ires Cre activity (red) does not colocalize significantly with AVP immunoreactivity (green) in the PVH. Images were taken at 20X magnification.

Mice carrying Cre-dependent expression of diphtheria toxin receptor (DTR) were purchased from the Jackson Laboratory (Gt(ROSA)26Sor^tm1(HBEGF)Awai^/J, item number: 7900, named *Rosa26^iDTR/iDTR^*. Study subjects were generated by mating *Rosa26^iDTR/iDTR^* mice with *Oxytocin-Ires-Cre* mice, from *Oxytocin-Ires-Cre:Rosa26^iDTR/+^* mice and their littermate control group *Rosa26^iDTR/+^* mice were generated. Since study subjects are littermates, potential genetic variation is equally distributed to study groups. To visualize *Oxytocin-Ires-Cre* expression, *Oxytocin-Ires-Cre* mice and *Oxytocin-Ires-Cre:Rosa26^iDTR/+^* mice were crossed with B6.Cg-Gt(ROSA)26Sor^tm9(CAG-tdTomato)Hze^/J mice (Ai9 mice, Jackson Laboratory, Bar Harbor, ME) to generate *Oxytocin-Ires-Cre:Ai9* mice and *Oxytocin-Ires-Cre:Rosa26^iDTR/+^Ai9* mice in which Cre expression can be directly visualized by tdTomato, a variant red fluorescence protein (RFP), using fluorescent microscopy [25]. Mice were housed at 22°C–24°C with a 12 hr light/12 hr dark cycle with food and water provided ad libitum. To achieve specific oxytocin neuron ablation, we used a strategy similar to that used previously for hypothalamic neuron ablation. Briefly, *Oxytocin-Ires-Cre:Rosa26^iDTR/+^* mice around 9 weeks of age were injected intraperitoneally with diphtheria toxin (DTX; 40 ng/g bodyweight); a second dose was administered one week later. Control *Rosa26^iDTR/+^* mice also received the same DTX treatment. All animals and procedures were approved by the animal welfare committee of the University of Texas Health Science Center at Houston.

**Figure 2:**
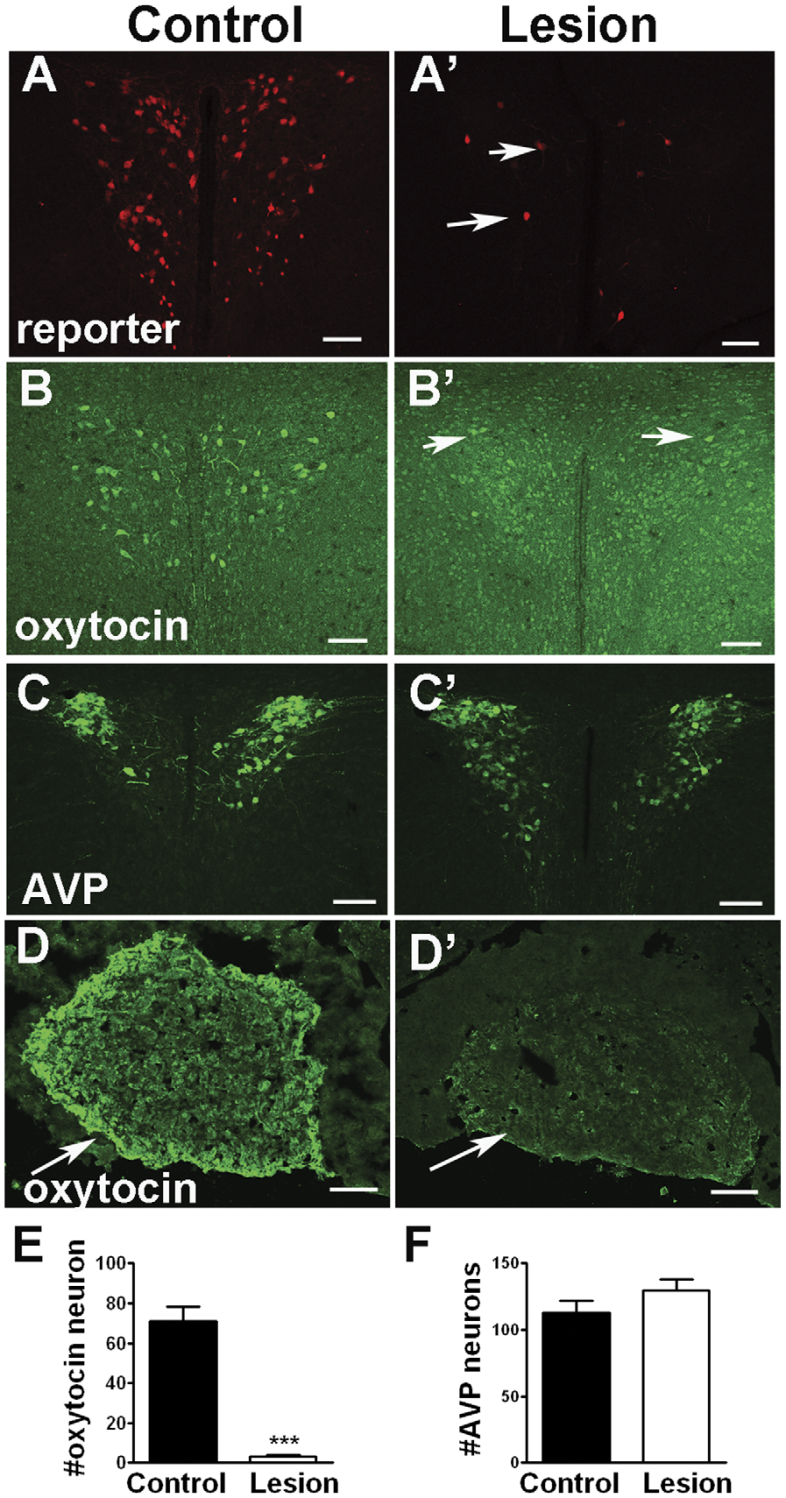
Specific lesion of oxytocin neurons. A and A′: Cre-dependent reporter RFP expression in *Oxytocin-Ires-Cre:Ai9* mice (control, A) and *Oxytocin-Ires-Cre:Rosa26^iDTR/+^:Ai9* mice (lesion, A′) after DTX administration. While numerous RFP positive neurons in control mice, reminiscent of oxytocin neurons, only a few positive neurons in lesion neurons (Arrows in A′). B and B′: Immunostaining for oxytocin in *Oxytocin-Ires-Cre:Ai9* mice (control, B) and *Oxytocin-Ires-Cre:Rosa26^iDTR/+^:Ai9* mice (lesion, B′) after DTX administration. While numerous oxytocin positive neurons in control mice, negligible number of oxytocin positive neurons in lesion neurons (Arrows in B′). C and C′: Immunostaining for AVP in *Oxytocin-Ires-Cre:Ai9* mice (control, C) and *Oxytocin-Ires-Cre:Rosa26^iDTR/+^:Ai9* mice (lesion, C′) after DTX administration. Similar patterns of AVP immunostaining were observed in both genotypes. D and D′: Immunostaining for oxytocin in the posterior pituitary of *Oxytocin-Ires-Cre:Ai9* mice (control, D) and *Oxytocin-Ires-Cre:Rosa26^iDTR/+^:Ai9* mice (lesion, D′) after DTX administration. While intensive oxytocin positive projections were observed in control mice (arrow in D), weak immunostaining was observed in lesion mice (Arrows in D′). We counted oxytocin and AVP positive neurons from matched PVH sections from each genotype (n = 3 each). The number of oytocin neuron was dramatically reduced (E) while AVP neuron number remained comparable (F). ***: p<0.001 using two tailed Student’s T test. Scale bars, 100 µm.

**Figure 3:**
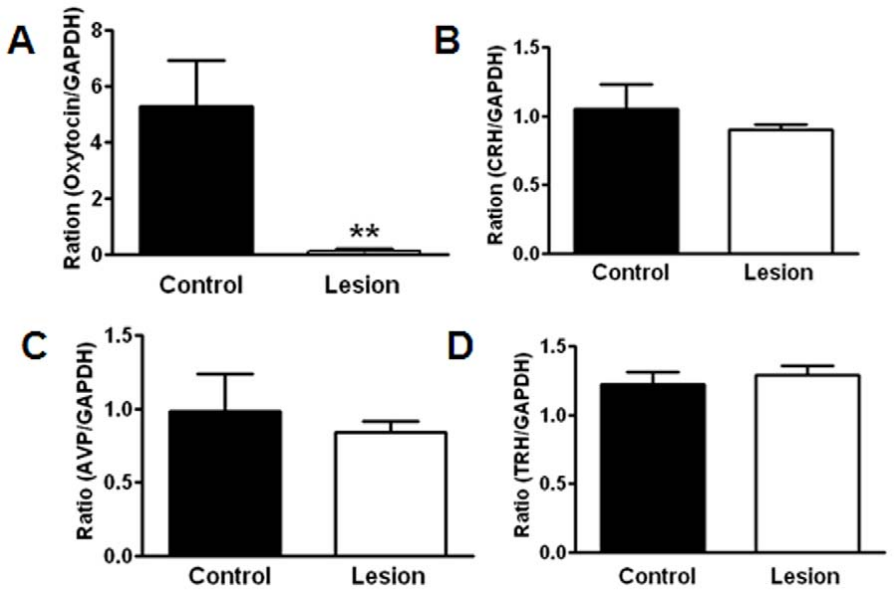
Expression of transcripts of PVH neuropeptides. Hypothalamic tissues were punched out from 10 weeks old mice of *Oxytocin-Ires-Cre:Rosa26^iDTR/+^* mice (lesion, n = 6) and *Oxytocin-Ires-Cre* mice (control, n = 7) that received DTX injections two weeks before. Expression of mRNA assessed by Q-PCR assays with GAPDH as an internal control using the punched tissues were shown for oxytocin (A), AVP (B), CRH (C) and TRH (D). **P<0.01 using two tailed Student’s T test.

### Body Weight Studies

Weekly body weight was monitored in *Rosa26^iDTR/+^* mice (or control mice after DTX treatment) and *Oxytocin-Ires-Cre:Rosa26^iDTR/+^* mice (or lesion mice after DTX treatment) fed standard mouse chow (Teklad F6 Rodent Diet 8664, Harlan Teklad, Madison, WI) from 4 to 20 weeks of age. For the high-fat diet study, a separate cohort of control and lesion mice were switched from chow diet to high fat, high sucrose diet (HFD, D12331 from Research Diets, NJ) from 10 weeks of age and maintained on HFD for 12 weeks. Body weight was monitored weekly. Body composition was measured at the end of study (22 weeks of age) using an Echo-MRI machine.

**Figure 4:**
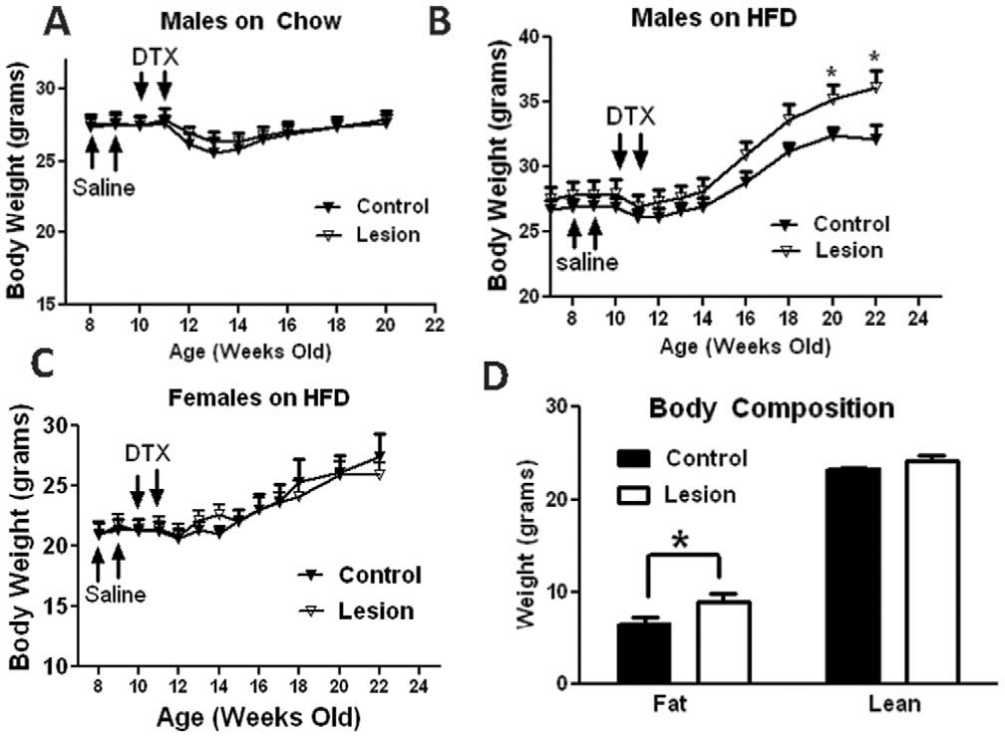
Body weight homeostasis in control and lesion mice. For body weight studies, *Oxytocin-Ires-Cre:Rosa26^iDTR/+^:Ai9* mice (lesion) and *Oxytocin-Ires-Cre:Ai9* mice (control) were first received two doses of saline treatment, each at 8 and 9 weeks of age, and then 2 doses of DTX, each at 10 and 11 weeks of age. Weekly body weight of these mice was measured up to 22 weeks of age on males fed chow (A, n = 7–8), males fed HFD (B, n = 6–8) and females fed HFD (C, n = 9–10). D. Body composition was determined at 22 weeks of mice shown in B. *p<0.05, two tailed Student’s T test.

### Energy Expenditure and Food Intake Measurements

Energy expenditure was assessed by measuring oxygen consumption with indirect calorimetry. Individually housed control and lesion mice maintained on chow diet at 12 weeks of age were placed at room temperature (22°C–24°C) in chambers of a Comprehensive Lab Animal Monitoring System (CLAMS, Columbus Instruments, Columbus, OH). Food and water were provided *ad libitum*. Mice were acclimated in the chambers for 48 hr prior to data collection. Oxygen consumption was first measured using mice fed chow for 2 days followed by another 2 days on HFD. Average O2 consumption was calculated for chow diet and HFD, and compared between genotypes. Daily food intake was measured for 1 week using mice that were individually housed for at least 1 week prior to assessment. Daily food intake was calculated from averaged food intake across the week.

**Figure 5:**
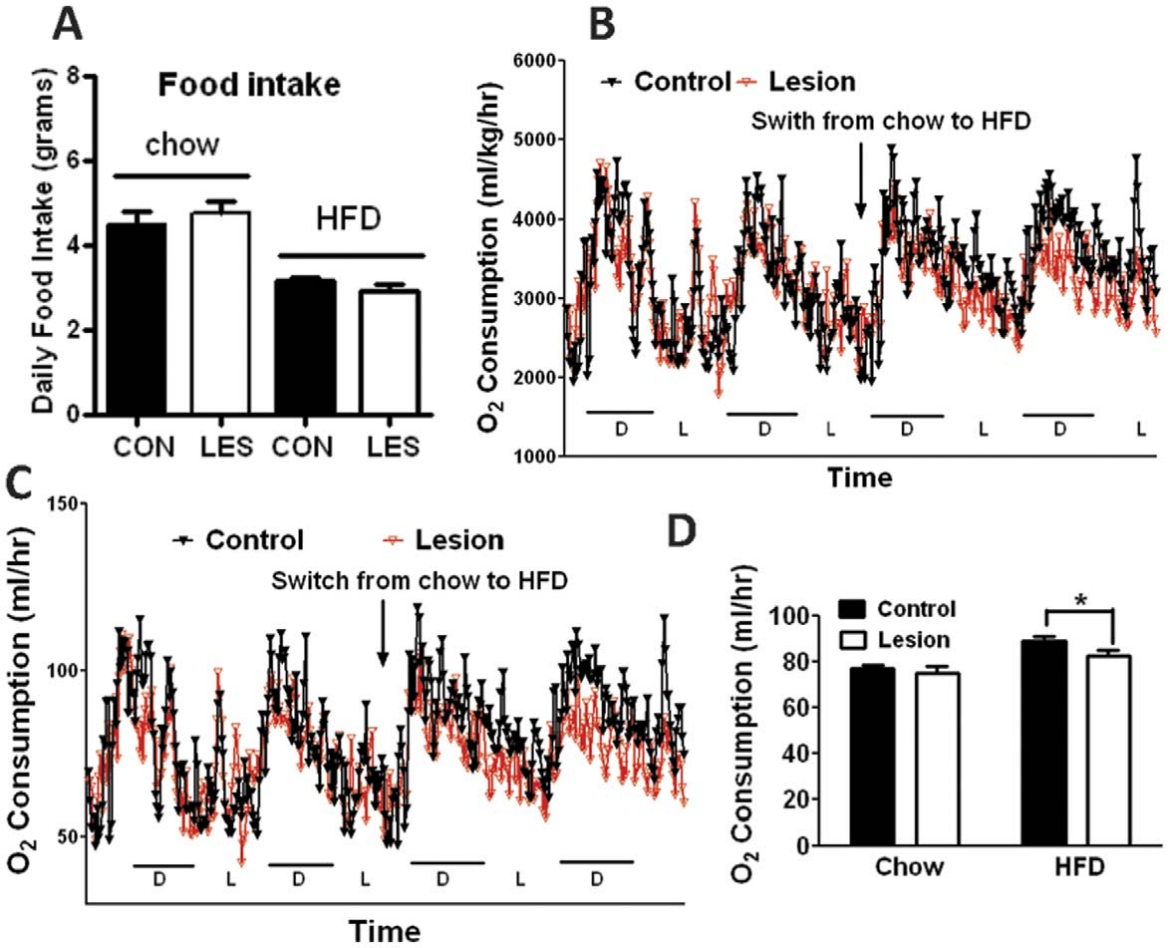
Food intake and energy expenditure in control and lesion mice. A. Daily food intake measured as average of 7-day consecutive daily food consumption of males fed chow and HFD at 14–15 weeks of age shown in Fig. 3A and B. Oxygen consumption was measured for male mice during 2 days on chow following by 2 days on HFD (n = 6−7 each), and the oxygen consumption of both genotypes was analyzed based on body weight (B) and on individual animal (C). D. O2 consumption across chow and HFD periods shown in C was averaged and compared between genotypes. *p<0.05, two tailed Student’s T test.

### Immunohistochemistry (IHC) Assays

For IHC experiments, free-floating brain sections were rinsed with PBS (pH 7.4) containing 0.1% Triton X-100 for 30 minutes, followed by the blocking in PBS containing 5% normal goat serum (Thermo scientific, Rockford, IL) and 0.3% Triton X-100 for 1 hour at room temperature. The sections were then incubated with polyclonal rabbit anti-OXYTOCIN (1∶1000, Phoenix), or polyclonal anti-AVP (rabbit 1∶500, Sigma or guinea pig 1∶1000, Phoenix) in PBS containing 2% normal goat serum and 0.2% Triton X-100 overnight at 4°C, respectively. For IHC on pituitary, pituitary tissues were taken out and post-fixed in 4% formalin overnight. Pituitary sections at 10 µM thickness were cut using a crytostat (Leica, Germany) and IHC was performed as described above on floating sections using the anti-AVP antibody. All sections were visualized and photographed with a TCS SP5 confocal microscope (Leica, Germany). To quantify cell numbers, in each mouse (n = 3−4), three sections at corresponding rostrocaudal levels (Bregma levels −0.58, −0.82 and −0.94 mm) were chosen. All immune-positive cells with clear profile were counted and the numbers from all animals were summed and then averaged as the number per section.

**Figure 6:**
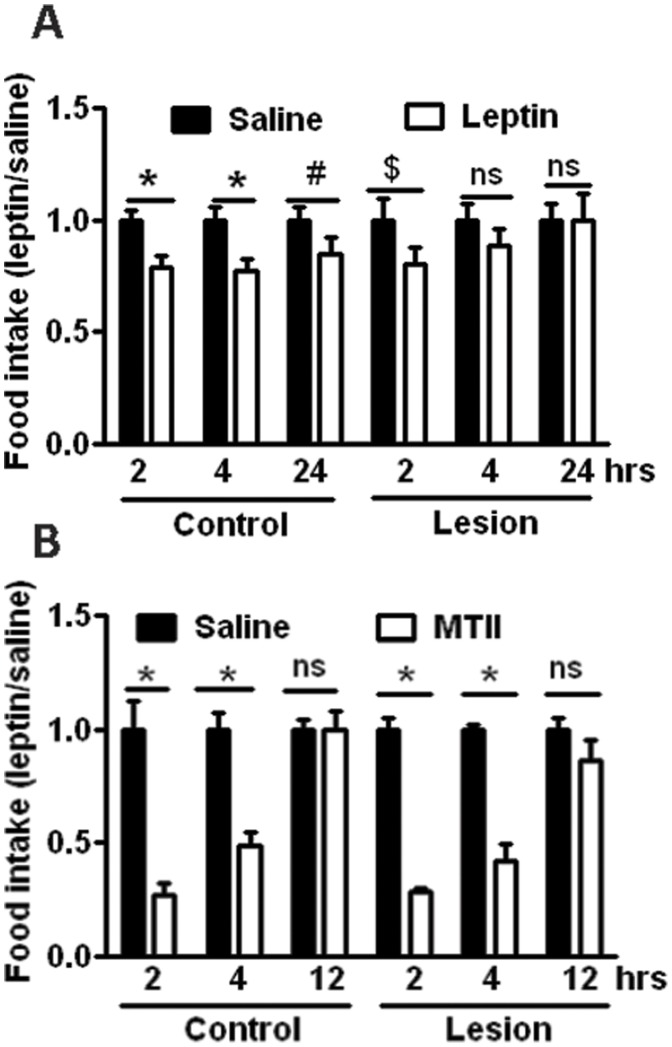
Food intake response to leptin and MTII. Control and lesion mice (n = 9 each) were treated with saline and DTX as in Fig. 3. All animals were singly housed for at least 1 week before feeding experiments. A. After overnight fasting, half of animals received saline and the other half leptin treatment, and one week later the same feeding experiment was repeated but in a crossover fashion. Food intake with leptin treatment was normalized and compared to that with saline treatment at 2, 4 and 24 hour periods. *p<0.05; #p = 0.082; $p = 0.069; ns, p>0.05 using paired Student’s T test. B. Food intake measurement was started at the onset of the dark cycle (8 pm) after 2-hour food deprivation. The MTII administration and data analysis was conducted in the same fashion as described in A. *p<0.05, ns, p>0.05 using paired Student’s T test.

### Leptin and MTII Effects On Food Intake

For leptin feeding experiments, a protocol similar to that previously described was used [26]. Individually housed male mice (11 wks) were acclimatized to daily injections of 200 µl of saline solution for 5 consecutive days before drug treatment. The day before experiment, mice were fasted overnight. After that, each mouse received either a saline or an intraperitoneal (i.p.) injection of recombinant murine leptin (Dr. F. Parlow, NHPP, 4.0 µg/g). Cumulative food intake and body weight was measured 2, 4 and 24 h after injection. After one week of recovery, the same protocol was performed in a cross-over fashion: the mice previously treated with saline received leptin and vice versa. The effect of leptin on food intake was calculated by normalizing food intake after leptin treatment to that observed after saline for each individual animal; this ratio was then averaged within each genotype.

MTII was purchased from Bachem (Torrance, CA). Male control and lesion mice on a chow diet, aged 12 wks, were housed individually for 5 days. Three days before the experiment the mice were habituated to daily i.p. injections using sterile saline. The experiment was performed on 2 separate days (2 days washout). On the day of the experiment, food was removed 4 h before the onset of the dark cycle (1400 h). At the onset of the dark cycle (1800 h), mice were injected i.p. with saline or 5 mg/kg MTII in saline. Cumulative food intake and body weight was measured 2, 4, and 24 h after injection.

### Quantitative PCR Assay

RNA was extracted from hypothalamic micro-punches using the Trizol Reagent (Invitrogen), reverse transcribed with RETROscript (Ambion) and amplified using SYBR green technology (Bio-Rad, Hercules, CA). The primers for oxytocin are 5′-CAAGAGGGCTGTGCTGGACCTGGATAT-3′ and 5′-GCGGGGTCTGTGCGGCAGCCA-3, for AVP are 5′-CTCCGCTTGTTTCCTGAGCCTGCTG -3′ and 5′-AGCAGCGTCCTTTGCCGCCCGG-3′, for TRH are 5′-GATGGCTCTGGCTTTGATCTT-3′ and 5′- GATCTATGAACCTCCGGCCT-3′, and for CRH are 5′-CATGCGGCTGCGGCTGCTGGTGT-3′ and 5′-GCGGCGCTCGGGGGACGGAT-3′. Assays were linear over five orders of magnitude.

### Statistical Analyses

Data sets are presented as mean ± SEM and analyzed for statistical significance using PRISM (GraphPad, San Diego, CA) for appropriate Student’s t tests. A *P* value of <0.05 was required for significance.

## Results

To achieve specific targeting of oxytocin neurons using Cre-loxP technology, we generated mice with Cre expression selective to oxytocin neurons. To ensure that Cre expression matches that of oxytocin, we employed a knock-in strategy and placed a DNA cassette containing an internal ribosomal sequence (IRES), Cre coding sequence and polyadenylation sequence directly after the stop codon of the endogenous oxytocin gene using homologous recombination (Fig. 1A). In this configuration, Cre and oxytocin are transcribed as a single mRNA and the IRES sequence allows simultaneous translation of both oxytocin and Cre proteins from the same mRNA, thus ensuring the co-expression of oxytocin and Cre. To confirm appropriate co-expression, we crossed mice harboring *Oxytoin-Ires-Cre* with a Td-tomato reporter strain which expresses Td-Tomato in a Cre-dependent manner. Using brain sections from the double transgenic mice (*Oxytocin-Ires-Cre:Td-tomato*), we performed immunostaining for oxytocin. We found a near complete colocalization of dsRed(red) and oxytocin (green) only in the PVH and the supraoptic nuclear, where oxytocin is known to be expressed (Fig. 1B). Approximately 92% of cells containing oxytocin immunoreactivity also expressed Cre activity in the PVH (n = 2). Similar patterns of colocalization were also seen using other Cre-dependent fluorescent reporter strains (data not shown). A few dsRed-positive neurons representing Cre expression appear not to express oxytocin in the overlay (Fig. 1B, arrows). This may reflect the fact that oxytocin expression in these neurons is too low for detection by immunostaining as it has been shown before that the number of oxytocin-expressing neurons depends on physiologic status [3]. In addition, almost all neighboring AVP neurons in the PVH are devoid of Cre activity in the Oxytocin-ires Cre mice (Fig. 1C), further suggesting that nearly all Cre activity is limited to oxytocin neurons. Thus, we have successfully generated *Oxytocin-Ires-Cre* mice, in which Cre expression nearly completely matches that of oxytocin. This strain can therefore be used for specifically targeting oxytocin neurons.

To achieve specific lesion of oxytocin, we crossed *Oxytocin-Ires-Cre* mice with *Rosa26^iDTR/+^* mice to generate *Oxytocin-Ires-Cre:Rosa26^iDTR/+^* mice. *Rosa26^iDTR/+^* mice express DTR, which is absent in mice, in a Cre-dependent manner. Upon administration of diptheria toxin (DTX), cells with DTRs take up DTX, which then causes cell death [27]. This approach has been successfully used for the lesion of neurons and other cell types [22], [28]–[29]. To efficiently detect the Cre-expressing neurons, we further crossed *Oxytocin-Ires-Cre:Rosa26^iDTR/+^* mice with a Cre-reporter line, Ai9 mice, which express ds-red with strong red fluorescence in a Cre-dependent manner [25]. To lesion oxytocin neurons in adulthood, we injected DTX (40 ng/g) by i.p. to *Oxytocin-Ires-Cre:Rosa26^iDTR/+^* mice (lesion) and *Rosa26^iDTR/+^* mice mice (control). Two weeks after injection, while we saw numerous neurons with dsRed-signal, representing Cre-expressing oxytocin neurons in control mice (Fig. 2A), we were only able to observe a few neurons in lesion mice (Fig. 2A′), suggesting efficient ablation of Cre-expressing neurons. To directly confirm the killing of oxytocin neurons, we performed immunostaining for oxytocin. As expected, we saw numerous oxytocin neurons in the PVH (Fig. 2B) and the supraoptic nucleus (not shown) of control mice. However, only a few oxytocin neurons can be observed in the PVH (Fig. 2B′) and the SON (not shown) of lesion mice (Fig. 2B′). To assess the degree of oxytocin lesion, we counted the number of oxytocin positive neurons. Whereas there were around 70+/−5 oxytocin positive neurons in a control PVH field, only 3+/−1oxytocin positive neurons were observed in a similar area in lesion mice (Fig. 2E, n = 3 mice). To determine whether the lesion is specific to oxytocin neurons, we performed immunostaining for vasopressin (AVP), which is expressed in a subset of neurons located in close proximity to oxytocin neurons. We observed a similar expression pattern of vasopressin neurons in control (Fig. 2C) and lesion mice (Fig. 2C′). When counted, the numbers of vasopressin neurons are similar between the treatments (Fig. 2F). Consistent with dramatic reduction in number of oxytocin neurons, oxytocin positive fibers in the posterior pituitary, the major projection site of oxytocin neurons was also dramatically reduced in lesioned mice (Fig. 2D′), compared to control mice (Fig. 2D). To further verify lesion of oxytocin neurons, we examined oxytocin expression in the hypothalamus and oxytocin levels in the blood. Using hypothalamic tissues obtained from mice 2 weeks after last DTX injection, oxytocin mRNA was severely reduced in lesioned mice compared to control mice (Fig. 3A). In agreement with our cell counts, AVP mRNA expression in the hypothalamus was not different between control and lesioned mice (Fig. 3B). Furthermore, mRNA levels in the hypothalamus were comparable between control and lesioned mice for both corticotropin releasing hormone (Fig. 3C) and thyrotropin releasing hormone (Fig. 3D). Thus, we have achieved efficient and specific lesion of oxytocin neurons in adult mice.

Cre expression alone can sometimes cause a difference in baseline body weight. To control for the potential effect of *Oxytocin-Ires-Cre* expression on body weight, we monitored body weight of *Oxytocin-Ires-Cre:Rosa26^iDTR/+^* mice and its control *Rosa26^iDTR/+^* mice up to 8 weeks of age. There was no difference in body weight between genotypes in males or females on chow (data not shown). In addition, to ensure a stable body weight with drug treatments, we administered saline once a week at the ages of 8 and 9 weeks before DTX administration. We found that the body weights of both groups remained stable and comparable during 2 weeks of measurement in males and females (Fig. 4 A–C). These data demonstrate that *Oxytocin-Ires-Cre:Rosa26^iDTR/+^*and *Rosa26^iDTR/+^* mice have the same baseline body weight. At 9 weeks of age, we administered DTX as described in Fig. 2. Following DTX administration, we monitored weekly body weights of control and lesion groups fed chow diet. Compared to the control group, lesioned male (Fig. 4A) and female (data not shown) mice exhibited similar body weight through 20 weeks of age, suggesting that oxytocin neurons are dispensable for body weight regulation on standard chow diet.

Mice lacking oxytocin or oxytocin receptor display late onset obesity with normal food intake [17], [20]. This suggests a defect in energy expenditure. To assess this possibility, we used high fat diet as a physiologic challenge for mice lacking oxytocin neurons. The animals were treated as described for the chow diet, but switched to high fat diet upon completion of DTX administration. Weekly body weights of lesion and control groups were measured through 22 weeks of age. In males, compared to the control group, lesion mice exhibited a comparable body weight up to 14 weeks of age, and then slowly developed higher body weight (Fig. 4B). At 22 weeks of age, lesion mice had body weight of (36.1+/−1.3 grams) while control group had body weight of (32.0+/−1.1 grams). In contrast, in females, lesioned mice exhibited no difference in body weight on HFD up to 22 weeks of age (Fig. 4C). These data suggest that oxytocin neurons are required for normal body weight regulation in response to HFD feeding in males, yet are dispensable in females. Since only lesioned males showed a body weight phenotype on HFD, we focused primarily on males in the following experiments.

To characterize the higher body weight in lesioned mice, we measured the body composition of lesioned and control mice fed HFD at 22 weeks of age (Fig. 4B). While lean masses of the different genotypes are comparable, fat mass is significantly higher in lesioned mice than in controls (Fig. 4D). To examine whether oxytocin neuron lesion affects feeding, we measured food intake of control and lesion mice on both chow and HFD. Average daily food intake over a one week period was comparable between genotypes on both chow and HFD (Fig. 5A), suggesting that oxytocin neurons are dispensable for food intake regulation on both chow and HFD. Given the difference in body weight between high fat diet treated groups but no difference in food intake, we next examined whether oxytocin neuron lesion alters energy expenditure in response to high fat diet challenge. We subjected mice to O2 consumption measurement using metabolic chambers (comprehensive lab animal monitoring system, CLAMS, Columbus, Ohio). We used cohorts of weight-matched mice at 14–15 weeks of age treated with DTX at 9 and 10 weeks of age, and there was no difference in body weight between genotypes. The animals were first measured for O2 consumption on chow for 2 days followed by another 2 days on HFD, as previously described [23]. When normalized to body weight, there was no difference in O2 consumption between genotypes during the chow period; however, O2 consumption appeared to be lower in lesioned mice compared to control mice during the HFD period, especially during the dark cycle (Fig. 5B). To rule out the possibility that lower O2 consumption is due to a difference in body weight or composition [30]., we also analyzed the data based on individual animal and saw a similarly lower O2 consumption in lesion mice on HFD (Fig. 5C). Indeed, statistical analysis showed that O2 consumption on HFD was significantly lower in lesioned mice compared to control mice; on a standard chow diet there was no difference in oxygen consumption between the groups.(Fig. 5D). Taken together, these data suggest that oxytocin neurons are required for the stimulation of energy expenditure in response to HFD feeding.

A previous study on lesioned AgRP neurons suggests the possibility of functional compensation in response to acute lesion [31]. To examine the possibility that acute lesion of oxytocin neurons induces functional compensation in feeding circuits and thereby masks a feeding effect in mice with oxytocin lesion, we tested the feeding response to leptin and MTII, an agonist of the melanocortin receptors. We used a cohort of control and lesion mice with comparable body weights. After overnight fasting, leptin administration significantly reduced 2- and 4-hour feeding in control mice by 25%, and also blunted 24-hour feeding by 20% (although this difference between treatment groups didn’t reach the level of significance). However, in lesion mice, leptin only produced 25% reduction of 2-hour feeding and the effects of leptin on 4- and 24-hour feeding are not significant between treatment groups (Fig. 6A). These results suggest that oxytocin neuron lesion leads to a blunted response to pharmacologic leptin action on feeding. For MTII, we examined its effect on feeding in the dark cycle, as previously established [1]. MTII potently reduced food intake to a similar degree in both control and lesion mice at 2- 4- and 12-hours post injection (Fig. 6B), suggesting that oxytocin neurons are dispensable for MTII mediated effects on feeding.

## Discussion

Compelling pharmacologic evidence suggests that oxytocin is anorexigenic, yet genetic models of oxytocin or oxytocin receptor deficiency display normal food intake with reduced energy expenditure [3]. To investigate this discrepancy, this study was designed to examine the physiological function of oxytocin neurons through specific oxytocin neuron lesion in adulthood. This approach eliminates the possibility of developmental compensation potentially associated with gene deletion during early embryonic stages in available models of oxytocin or oxytocin receptor deficiency. Using *Oxytocin-Ires-Cre* mice generated in this study, we have achieved specific lesion of oxytocin neurons through a combination of Cre-mediated expression of DTR in oxytocin neurons and adult DTX administration. This DTR-based lesion approach has been used by others to effectively lesion POMC and AgRP neurons [22], [28]. Our lesion targeted more than 95% of oxytocin neurons with a high degree of specificity, as there was no reduction in nearby AVP neurons or levels of CRH or TRH mRNA after oxytocin neuron lesioning. This suggests that our *Oxytocin-Ires-Cre* mice express Cre selectively in the vast majority, if not all, of oxytocin neurons and will be useful in future studies targeting oxytocin neurons for genetic manipulation.

Our results showed that adult lesion of oxytocin neurons had no effect on body weight, food intake or energy expenditure on chow. Previous results using mice with deficiency of oxytocin or its receptor showed evidence for reduced energy expenditure on chow [17], [20]. The difference between these studies and ours may be related to different genetic backgrounds between study subjects, housing conditions or different diets used. Evidence for the potential importance of dietary differences stems from our results showing that mice that lack oxytocin neurons develop obesity on HFD with normal food intake but reduced energy expenditure. The selective effect on energy expenditure is consistent with the metabolic effects associated with deficiency of oxytocin or its receptor, both of which also exhibit selective reduction in energy expenditure, but not food intake [17], [20]. In addition, oxytocin neurons are anatomically positioned to regulate energy expenditure. Oxytocin neurons are located in the PVH and the PVH has been implicated in energy expenditure regulation through projections to the raphe pallidus or to the spinal cord, which control sympathetic output to the brown fat tissues, the major thermogenic organ in rodents [2], [32].

Interestingly, despite the limited affect on physiological food intake on chow or HFD, oxytocin neuron lesion led to a blunted response to the anorexigeic effect of leptin. This result suggests that oxytocin neurons at least partly mediate leptin action on feeding and that functional compensation for leptin action occurs in response to inducible loss of oxytocin neurons. It has been shown previously that adult lesion of AgRP neurons produces a starvation phenotype, which is averted in the setting of leptin-deficient obesity [31]. This result suggests that rapid functional compensation from redundant pathways exist and are sufficient to ameliorate the detrimental effects of acute neuron loss or dysfunction. Given the biological importance of food intake regulation, it is plausible that oxytocin neurons represent just one subset of downstream neurons that mediate leptin action on food intake. In the setting of oxytocin neuron loss or dysfunction one might imagine that non-oxytocin, leptin-responsive pathways assume the function of the “lost” oxytocin neurons and restrain food intake. For example, in the absence of oxytocin, AVP can bind and activate oxytocin receptors [33] and AVP is up-regulated in response to salt challenging [34]. These results suggest that AVP can be one of the candidates that compensate for oxytocin neuron function. In support of this, the AVP pathway has previously been shown to be involved in feeding regulation [35]. Of note, mRNAs of AVP and other known neuropeptides in the PVH remain at comparable levels in oxytocin neuron lesioned mice relative to controls, suggesting that these neuropeptides cannot compensate for oxytocin neuron ablation at the transcription level under these experimental conditions. Nonetheless, the blunted feeding response to leptin following oxytocin neuron ablation suggests that redundant pathways are not sufficient to fully compensate in response to acute pharmacologic leptin administration. In this regard, it is important to note that the observed effects of oxytocin on feeding are largely based on pharmacologic studies [9], [13]–[14], [36]. On the other hand, the deficits in high fat diet induced energy expenditure observed in this experimental paradigm suggest a less “plastic” neural pathway regulating diet induced energy expenditure that is unable to compensate for the acute loss of oxytocin neurons. Thus, our results suggest that oxytocin neurons are involved in both energy expenditure and food intake. Specifically, oxytocin neuron-mediated energy expenditure is required for response to high-fat feeding while their function in food intake can be masked by redundant pathways under normal conditions but may be revealed by acute pharmacologic approaches. These results provide an explanation for the discrepancy between results from pharmacological studies suggesting a selective role of oxytocin in feeding and those from genetic studies suggesting a selective role in energy expenditure.

Oxytocin neuron lesion had no effect on melanocortin agonist inhibition of feeding, but produced a blunted response to leptin. Although somewhat unexpected, this finding supports the concept that melanocortin and leptin feeding circuits do not completely overlap. For example, GABAergic non-POMC neurons in the Arc may mediate leptin action on feeding through GABAergic action onto PVH neurons including oxytocin neurons [24], [37]. In addition, a recent report suggests that a small subset of PVH neurons express leptin receptors, which may directly engage oxytocin neurons in mediating leptin action on feeding [38]. The dispensable role of oxytocin neurons in MTII action on feeding is consistent with the result that oxytocin is dispensable for feeding inhibition by Cholecystokinin, which requires MC4R action [18], [39].

Surprisingly, our results suggest that the function of oxytocin neurons in body weight regulation is sexually dimorphic, i.e. oxytocin neurons are required for energy balance in males, but not in females. A literature survey indicates that previous studies on oxytocin in feeding and body weight were primarily focused on males. This result is consistent with the previous result that oxytocin receptor deletion leads to obesity only in males, but not in females [20]. It might be the case that in females oxytocin neurons are preferentially involved in other functions such as reproduction and stress. For example, oxytocin has been reported to have opposite roles in anxiety-related behaviors in males and females [40]. One study, however, demonstrated that oxytocin deficiency in females led to obesity in a similar fashion to males [17]. The discrepancy cannot be explained by different diets used since our lesion females had comparable body weights to controls on both chow and HFD. Whether it is due to difference genetic background or represents a variable associated with developmental compensation is unknown.

In summary, inducible lesion of oxytocin neurons in adult mice demonstrates that oxytocin neurons are required for diet induced energy expenditure but are not necessary for food intake regulation. Moreover, whereas oxytocin neurons are required for a full physiologic response to leptin administration, they are dispensable for the anoretic action of melanocortin agonists. This suggests that additional, non-oxytocin dependent neural circuits play important roles in the regulation of food intake.

## Acknowledgments

The authors would like to thank Dr. Junghun Song and Ms. Nandini Maharaj for technical help.
